# Pityriasis lichenoides et varioliformis acuta in a patient on zanubrutinib for marginal zone lymphoma

**DOI:** 10.1016/j.jdcr.2024.11.017

**Published:** 2024-11-27

**Authors:** Aliya Rogers, Rachel Lefferdink, Vida Ehyaee, Parul Goyal

**Affiliations:** a Department of Transitional Year, Ascension Resurrection Medical Center, Chicago, Illinois; b Department of Dermatology, Rush University Medical Center, Chicago, Illinois; c Department of Pathology, Rush University Medical Center, Chicago, Illinois

**Keywords:** Bruton’s tyrosine kinase inhibitor, cancer care, cutaneous toxicity, marginal zone lymphoma, oncodermatology, pityriasis lichenoides et varioliformis acuta, PLEVA, zanubrutinib

## Introduction

Bruton’s tyrosine kinase (BTK) has become an important therapeutic target in the treatment of B-cell malignancies. BTK is a critical component of the B-cell receptor signaling cascade. Inhibiting the kinase therefore affects B-cell proliferation, survival, migration, and immunoglobulin synthesis.^1^ Zanubrutinib, a second generation oral BTK inhibitor, was approved by the Food and Drug Administration in 2021 for adults with relapsed or refractory marginal zone lymphoma and has been found to be an effective treatment option for this patient population.^2^ We report a case of a patient with marginal zone lymphoma who developed pityriasis lichenoides et varioliformis acuta (PLEVA) likely triggered by the initiation of zanubrutinib therapy.

## Case report

**Fig 1:**
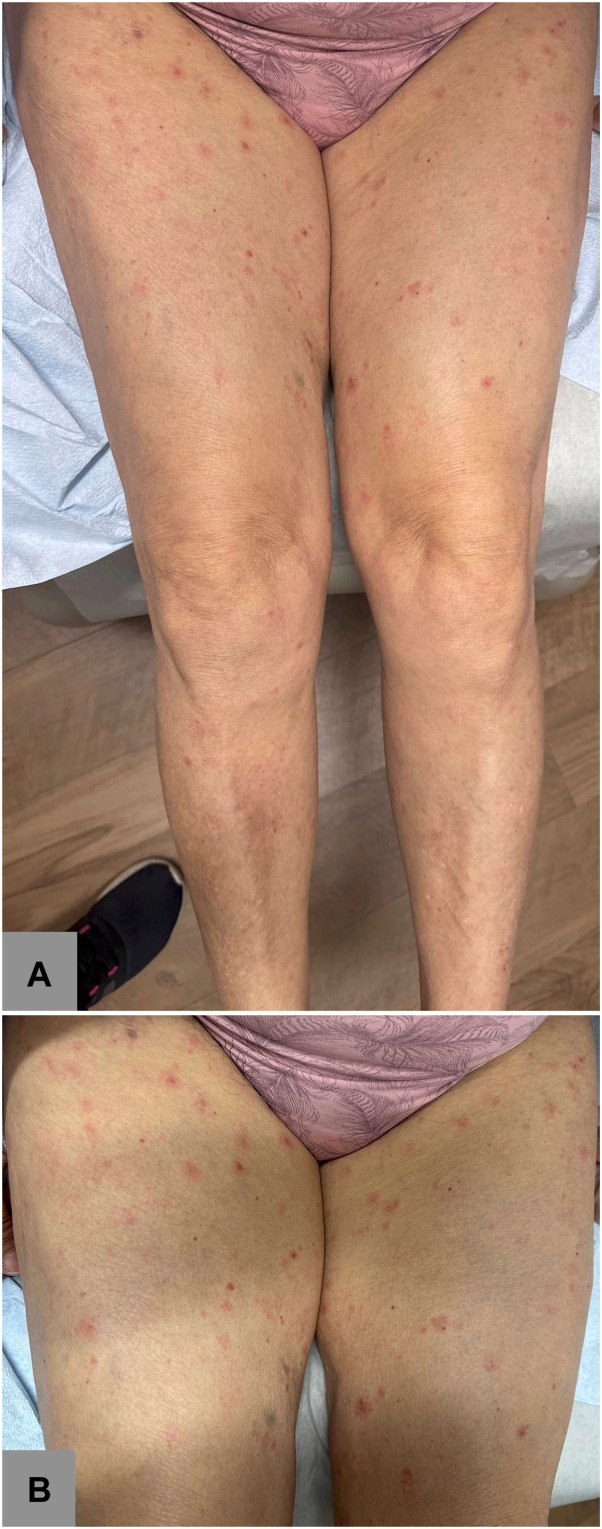
Numerous *pink*, indurated papules and *crusted brown* papulonodules with superficial lichenification and excoriation noted diffusely over the lower extremities (**A** and **B**).

A 74-year-old woman with a history of refractory marginal zone lymphoma (status post prior administration of rituximab in 2016, 2018, and 2019) was referred to our dermatology clinic by hematology/oncology for a rash on the lower extremities. The patient noted the eruption had developed shortly after starting zanubrutinib 160 mg twice daily 10 months prior to presentation, with no history of starting any other medications at that time. She had started zanubrutinib approximately 4 years after her last course of rituximab. She reported that the eruption began as red macules on the legs that then became brown, crusted papulonodules with slight superficial scale. The patient denied any pruritus or pain. She had been initially started on topical corticosteroids without significant improvement after 3 months of regular application. Physical exam revealed pink, indurated papules and crusted brown papulonodules with superficial lichenification and excoriation scattered on the lower extremities (Fig 1). A punch biopsy on the right thigh was performed for definitive diagnosis. The patient was instructed to continue topical corticosteroids twice daily pending pathology results.

**Fig 2:**
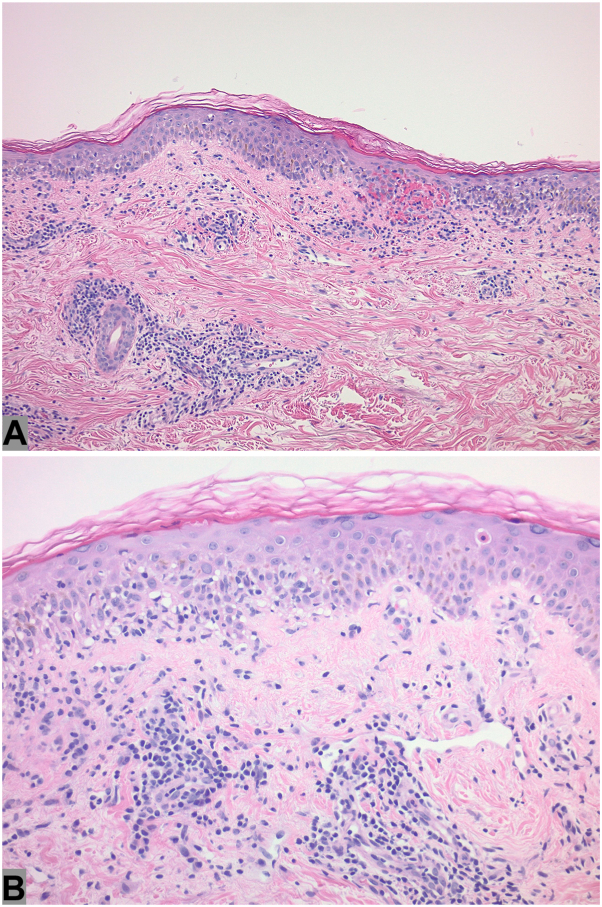
Histopathologic section revealing parakeratosis and vacuolar interface changes in the basal cell layer, accompanied by a lymphocytic infiltrate (interface dermatitis). There are moderately dense perivascular lymphocytic infiltrates in the superficial and mid-dermis (hematoxylin-eosin, ×200 (**A**), ×400 (**B**)).

Histology was notable for interface dermatitis with vacuolar degeneration of the basal cell layer, heavily infiltrated by lymphocytes. There was evidence of parakeratosis and a moderately dense perivascular lymphocytic infiltrate in the superficial and mid-dermis. Extravasated erythrocytes were also observed within the papillary dermis (Fig 2). These findings were consistent with a diagnosis of PLEVA, likely triggered by zanubrutinib initiation.

**Fig 3:**
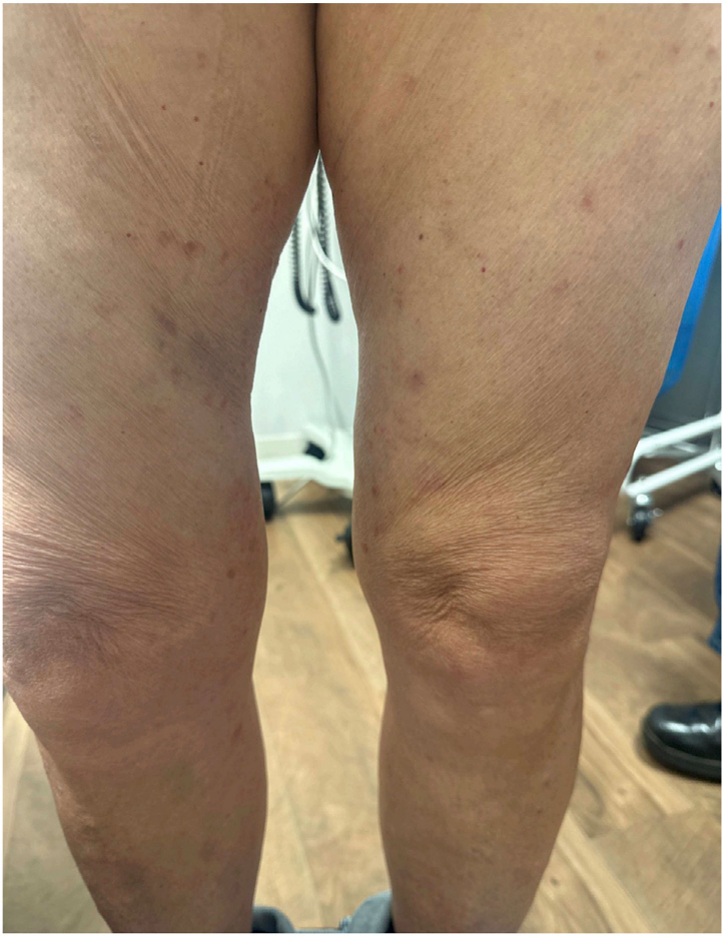
After treatment with doxycycline 100 mg twice daily for 3 months.

The patient was prescribed oral doxycycline 100 mg twice daily for 3 months. At a follow-up visit 3 months later, the patient had significant improvement with a reduction in lesions (Fig 3). She was then instructed to resume taking doxycycline for another 2 months to see if additional improvement could be achieved. She is currently completing the fourth month of therapy at this time.

## Discussion

PLEVA belongs to a group of inflammatory skin disorders termed pityriasis lichenoides. It presents as a spontaneous eruption of erythematous macules that evolve into papules with a fine scale that rapidly undergo hemorrhagic necrosis and ulceration with an overlying red-brown crust. The lesions may be asymptomatic or present with burning or pruritus. PLEVA tends to form on the trunk, extremities, and flexural areas but can also be generalized. The eruption is polymorphous and can last from weeks to years in a remitting and relapsing pattern. It may evolve into its chronic form, which is known as pityriasis lichenoides chronica.

The etiology of PLEVA is unknown. Three major hypotheses are discussed in the literature: (1) an inflammatory response to an infection, (2) an immune complex mediated hypersensitivity reaction, and (3) a T-cell lymphoproliferative etiology.^3^ The evidence for a T-cell lymphoproliferative process includes studies citing a predominance of cytotoxic CD8^+^ T-cells in the inflammatory infiltrate,^4^ presence of large atypical CD30^+^ cells,^5^ and detection of T-cell clones in biopsies.^6^

The lymphoproliferative hypothesis is further supported by cases that describe the development of pityriasis lichenoides after the use of medications that alter T-cell behavior. In one case, a 69-year-old man with diffuse large B-cell lymphoma was treated with tafasitamab, an anti-CD19 therapy, and developed pityriasis lichenoides chronica.^7^ In that case, the authors proposed that pityriasis lichenoides chronica may have developed due to T-cell dysregulation caused by the drug altering B-cell response. In another case, a 79-year-old man with multiple myeloma was treated with cevostamab, a drug that causes T-cell activation and proliferation, and developed PLEVA.^8^ In that case, the authors proposed that the rash may have been triggered by the drug’s effect on T-cell behavior.

Previously reported dermatologic toxicities of BTK inhibitors include bruising, rash, panniculitis, and skin infections, which have all been proposed to be related to off-target inhibition of the epidermal growth factor receptor.^1^^,^^9^ To our knowledge, this is the first case report of PLEVA triggered by zanubrutinib. In our case, development of pityriasis lichenoides may be related to the BTK inhibitor’s action on the B cell receptor signaling cascade causing an alteration in T cell activity. Additionally, BTK has been found to be expressed in T-cells, and BTK inhibition in vivo has been reported to increase the persistence of activated T-cells, decrease the Treg/CD4^+^ ratio, and elevate T-cell numbers and T-cell related cytokines.^10^ This aligns with the hypothesized lymphoproliferative etiology of PLEVA and could potentially explain why it developed in our patient shortly after zanubrutinib initiation.

Multiple cases in the literature report treatment with oral anti-inflammatory antibiotics, including tetracyclines and erythromycin, as the most effective first-line therapy for pityriasis lichenoides.^3^ Topical corticosteroids or topical immunomodulators can also be used as adjunctive therapy. If no response to first-line treatment, ultraviolet B or psoralen plus ultraviolet A light treatments have been reported as effective second-line therapies.^3^ For refractory disease, therapeutic options include methotrexate, acitretin, dapsone, and cyclosporine.^3^

Based on our patient’s medical history and clinical presentation, differential diagnosis included cutaneous T-cell lymphoma (especially lymphomatoid papulosis which clinically can appear similar to PLEVA^3^), cutaneous B-cell lymphoma, and pseudolymphoma. Therefore, obtaining a biopsy in this case was critical to confirm a diagnosis and rule out a potential malignancy.

## Conclusion

PLEVA is a benign entity of uncertain etiology that typically responds well to therapy with systemic antibiotics and/or phototherapy. We report this case of zanubrutinib-triggered PLEVA to increase clinician awareness of this rare potential side effect and to discuss the various therapeutic options.

## Conflicts of interest

None disclosed.
